# Re-evaluation of the interrelationships among the behavioral tests in rats exposed to chronic unpredictable mild stress

**DOI:** 10.1371/journal.pone.0185129

**Published:** 2017-09-20

**Authors:** Congli Hu, Ying Luo, Hong Wang, Shengnan Kuang, Guojuan Liang, Yang Yang, Shaoshan Mai, Junqing Yang

**Affiliations:** Department of Pharmacology, Chongqing Medical University, the Key Laboratory of Biochemistry and Molecular Pharmacology, Chongqing, China; Chiba Daigaku, JAPAN

## Abstract

The chronic unpredictable mild stress model of depression has been widely used as an experimental tool to investigate human psychopathology. Our objective was to provide an update on the validity and reliability of the chronic unpredictable mild stress model, by analyzing the interrelationships among the indexes using stepwise discriminant analysis and Pearson correlation coefficient to examine the possible combinations. We evaluated the depressive rats in both the presence and the absence of chronic unpredictable mild stress, using weight change, percentage of sucrose preference, coat state, splash test, open-field test, elevated plus-maze test, forced swimming test, and Morris water maze test. The results showed that 6-week-long chronic unpredictable mild stress produces significant depression and anxiety-like behavior. The combination of body weight change, percentage of sucrose preference, coat state score, open-field score, grooming latency of splash test, immobility time in force swimming test, and platform crossing in the Morris water maze test can effectively discriminate between normal and chronic unpredictable mild stress rats. Strong interrelationships were noted among these indexes in both open-field test and elevated plus-maze test. In conclusion, there might be certain criteria for the combination of behavioral endpoints, which is advantageous to more effectively and reliably assess the chronic unpredictable mild stress induced depression model.

## Introduction

Depression is a common chronic affective disorder. On an average, one in four women and one in six men suffer from depression during their lifetime [1], and up to 65% of the affected individuals have recurrent episodes of this disorder [2]. On account of the restriction of drawing experimental materials and the complicated etiology, animal models are an indispensable tool for the research on the mechanism of depression and antidepressant drugs. So far, there are more than 20 kinds of depressive animal models, among which the chronic unpredictable mild stress (CUMS) model, developed originally by Paul Willner in 1987 [3], is the most widely used. CUMS results in a state of reduction in sucrose intake (and/or preference) that is akin to the decrease in responsiveness to rewards, which is the foundation for anhedonia, a central feature of depressive disorders [4]. Since the inception of the CUMS model, considerable behavioral research has extended the behavioral endpoints of this model in different aspects, such as decrease in sexual and aggressive behaviors, increase in immobility in the forced swim test (FST) and learned helplessness, changes in sleep architecture, and decrease in self-care and grooming [5]. Almost all these depression-related effects are reported to co-exist alongside CUMS-induced anhedonia [6]. Moreover, many other studies have also reported behavioral endpoints other than anhedonia or the other depression-related effects mentioned above, such as alcohol preference, anxiety, exploration, locomotor activity, and memory [6], which might simulate some facets of human psychomotor retardation and neurocognitive deficit. A large number of studies have shown that mood disorders are always accompanied by cognitive dysfunction, for example, memory deficits and poor sustained attention [7,8]. In fact, CUMS rats have been reported to exhibit cognitive dysfunction in previous studies [9,10]. Considering the fact that each endpoint represents only one symptom of human body, researchers often adopt a combination of different behavioral endpoints, instead of using just sucrose consumption, to examine the validity of this model. Different research groups have different methods, for example, sucrose consumption + locomotor activity + behavioral despair [11], body weight + sucrose consumption + anxiety + locomotor activity [12], coat state + grooming behavior + locomotor activity [13], locomotor activity + behavioral despair + memory deficits + anxiety [14]. To date, there is no fixed criterion for combining these behavioral endpoints, and to the best of our knowledge, no study has analyzed the relationships between CUMS-induced anhedonia and other behavioral endpoints.

These issues led us to hypothesize that there are some indispensable endpoints that compose a definite criterion along with anhedonia for evaluating the CUMS model, and some unnecessary behavioral tests or indexes can be removed if any interrelationships among these indexes were discovered. To test this hypothesis, we established the CUMS model in rats, investigated the relationship between sucrose preference and other behavioral endpoints by conducting the most frequently used behavioral tests, adopted stepwise discriminant analysis, set up a distinguishing function model, and utilized the Pearson correlation coefficient to analyze the interrelationships among these ethological indexes. Through this study, we hope to establish the interrelationships among these endpoints and facilitate future research on depression.

## Materials and methods

### Animals

Sprague–Dawley rats (Male, 180–200 g; from the Experimental Animal Center of Chongqing Medical University) were initially housed in standard laboratory conditions (12 h light/dark cycle, lights on from 08:00 to 20:00; temperature = 22 ± 2°C; humidity = 50% ± 10%) with free access to food and water except during experimental procedures. All experiments in our study were conducted in accordance with the Chongqing Management Approach Guide to the Care and Use of Laboratory Animals (Chongqing Government Order No. 195). All experiments involving rats were reviewed and approved by the Animal Laboratory Administration Center and Ethics Committee of Chongqing Medical University [SYXK (Chongqing) 2012–0001].

### Preliminary work & general procedure

All rats were allowed to acclimatize to their new environment for two weeks following their arrival. Before formal experiment, picked out 80 rats whose horizontal scores in open-field test were between 30 and 120. The qualified animals were random divided into two groups. One group was housed individually in separate cage and subjected to chronic unpredictable mild stress (CUMS) (n = 40), the others were housed in the standard condition with food and water without any stress as controls (n = 40)for 6 weeks.

When the groups were assigned, the initial coat state was assessed. The body weight and sucrose preference were measured every week when the CUMS procedure was performed. Two days after the termination of CUMS, all the rats were subjected to the behavioral tests.

**Fig 1:**
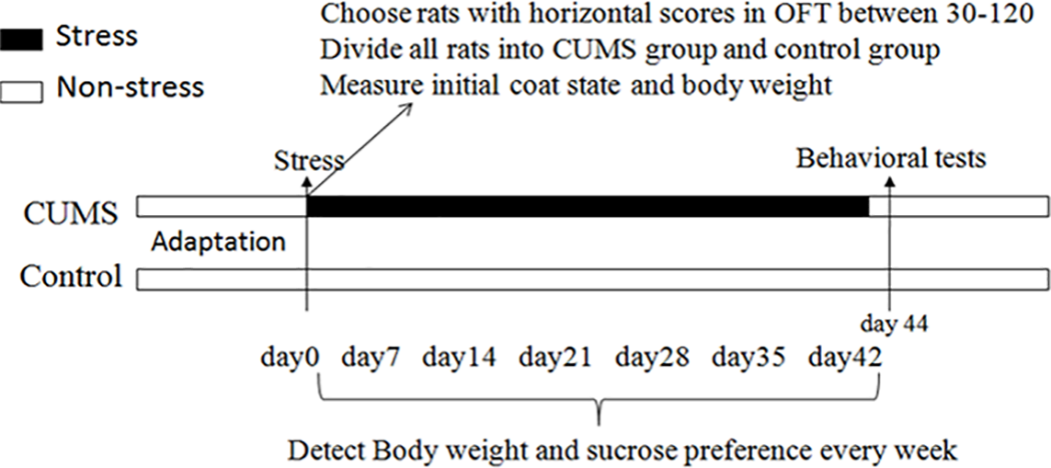
Preliminary work and general procedure of this study.

The general procedure is shown in **Fig 1**.

### Chronic unpredictable mild stress

This CUMS regimen was a minor variation of the protocol described previously [3]. The rats were chronically exposed to various randomly scheduled, low-intensity social and environmental stressors during 6 weeks of the procedure. These stressors consist of isolation (as a continuous social stressor), tail cramping (for 1 min), deprivation of food or water (for 24 h), forced swimming (4°C water, for 5 min), heat stimulation (45°C water, for 5 min), cage-tilting (45°,for 24 h), wet bedding (200 ml water per cage, for 24 h), removal of sawdust (for 24 h), reversal of day and night, glare flash (for 3 h) and loud noise (92 dB,1500 Hz, for 3 h). Rats received one of these stressors per day in **Table 1**. The same stressor was not applied in 3 consecutive days so that animals could not predict the occurrence of stimulation.

**Table 1:** CUMS schedule.

Stressors	Days of CUMS experiments
Isolation	1,15,27
Tail cramping for 1 min	2,16,25,36
Food deprivation for 24 h	3,19,37
Water deprivation for 24 h	10,20,31
Forced swimming 4°for 5 min	4,13,26,41
Haet stimulation 45°C for 5 min	5,21,29,40
Cage-tilting 45°for 24 h	6,17,38
Wet bedding for 24 h	8,18,30,39
Sawdust empty for 24 h	9,24,32
Reversal of day and night	7,14,28,35,42
Glare flash for 3h	12,22,34
Loud noise for 3h	11,23,33

### Behavioral tests

Animals were brought to the testing room approximately 30 min before the test, and all behavioral tests were performed under double-blind principle. All the rats were subjected to the behavioral tests and the order as follows:

#### Sucrose preference test (SPT)

The SPT was performed according to a previous study [3] with minor modifications, at 08:30 AM on days 7, 14, 21, 28, 35, and 42 during the CUMS phase. Before the first time of the test, all rats were trained to get accustomed to 1% sucrose solution(w/v) in a quiet environment.Two bottles of 1% sucrose solution were placed on each cage for 24 h. For the following period of 24 h, 1% sucrose in one bottle was replaced with pure water. After adaptation, rats were deprived of food and water for 24 h, followed by the SPT the next morning, in which the rats were fed with two pre-weighed bottles of liquid at the same time: the one containing 1% sucrose solution and the other pure water. The bottles were counterbalanced across the left and right sides of the cages throughout the experiment to avoid position preference effects. At intervals of 1 h, 12 h, and 24 h, the two bottles were re-weighed.

#### Coat state

Dirty coats in rats often represent low self-care behavior (e.g., unwillingness to self-clean). The observed coat state was evaluated with a quantitative scale that assessed eight different body parts: head, neck, dorsal coat, tail, forelimb, hindlimb, ventral coat, and genital region [13]. The total score of the coat status was obtained by attributing a score of 0 (clean coat) or 1 (dirty coat or in abnormal state) to each of the eight parts. The mean of the results was statistically analyzed.

#### Splash test

Splash test was performed in both control and CUMS-exposed rats 2 days after the coat state evaluation. Control animals were isolated for 1 day before testing, while the cages of the stressed rats were changed at the same time. This test was performed under a red light (15 W). An atomizer spray containing 10% sucrose solution was squirted on the dorsal coat of a rat in its home cage [13]. The sucrose solution dirties the coat and induces grooming behavior. After applying sucrose solution, the time spent grooming was recorded for a period of 5 min as an index of self-care and motivational behavior[15–17].The proportion of time spent in grooming (= grooming time / 5 min) was calculated.

#### Open-field test (OFT)

The OFT was performed according to Lin et al [18]. The inner surface of the test apparatus was painted with black.The floor of the apparatus (100 cm × 100 cm × 40 cm) was divided into 25 identical squares (20 cm × 20 cm) with white stripes. In a dark (visibility was 5 m) and quiet room, a single rat was placed at the center of the arena and allowed to explore for 5 min. We recorded the number of squares crosses by the rats (with all four paws crossing the line, each line cross was scored as 1 point), the rearing behaviors (each rearing was scored as 1 point), the grooming behaviors (each grooming was scored as 1 point), the feces (each piece of feces was scored as 1 point; the urine was also scored as 1 point), and the latency of locomotion. After each of these test, the excrements were cleaned and the arena was dealt with 75% alcohol. The score was calculated by the sum.

#### Elevated plus-maze (EPM) test

The EPM consisted of two open arms (50 cm × 10 cm) and two closed arms (50 cm × 10 cm, surrounded by 40-cm plastic walls) that originated from a common central platform (10 cm × 10 cm). The apparatus was elevated to a height of 50 cm above the floor. This test was performed in a dark (visibility, 5 m) environment and after OFT to avoid cowering of rats along the length of enclosed arms and to enhance the total amount of arm entries. The rat was placed on the central platform with the head facing towards an open arm and allowed to explore for 5 min [19,20]. The proportion of time spent in the open arms (= the time spent in the open arms / 5 min) and the number of entries into the open arms were calculated. The rat was considered as entering a new arm when it introduced its four paws in the arm. The maze was rinsed between sessions with 75% alcohol and dried with a towel.

#### Forced swimming test (FST)

The FST, also known as the behavioral despair test, is centered on a rodent's response to the threat of drowning, the results of which have been interpreted as a measure of susceptibility to negative mood [21]. Rats were forced to swim in a vertical plastic cylinder (diameter 30 cm,height 50 cm) containing 30 cm of water maintained at 25 ± 1°C. The rats were allowed to become used water cylinder during the first 2 min. Because of the fear of drowning, the rats would swim excitedly after placing into water. But no matter how actively strove, the rats had no way to escape from the cylinder, which made them fall into a despaired mood and manifested as floating on the surface of water.During the following 5 min, the duration of immobility time for every rat was measured by two observers who were blinded to the kind of treatment. The animal was dried with a towel and the water was changed after each swimming test. The proportion of time spent in the state of immobility was calculated.

#### Morris water maze (MWM) test

The Morris water maze apparatus consisted of a circular basin (diameter 180 cm, height 50 cm) which contained 24 cm of water maintained at 25 ± 1°C, with a clear escape platform (learning place, invisible condition, diameter 8 cm) placed 1 cm below the water surface[14]. Several visual cues surrounding the maze were available on the walls, and the observer stayed in the same place for each trial. The pool was equally divided into 4 quadrants: NW, NE, SE and SW. This test include 2 periods: initial spatial training and probe test.

Initial spatial training: Before being placed into the water, the animal was allowed to stand on the platform for 60 s to realize the existence of the hidden platform and memorize the environment. The rat was then placed into the water facing the mid -section of the wall at one of the four quadrants and allowed to swim freely until they found and climbed onto the platform. If the rat failed to locate the platform within 180 s, it was guided to the platform by observers and allowed to stand there for 10 seconds. Each rat was subjected to four trials per day, and the starting position was different for each trial. The interval between two operations of placing a given rat into water was at least 15 min. The spatial training was continuously conducted for 4 days.

Probe test: On the fifth day, the platform was removed. The rat was placed into the quadrant opposite to the one where platform had been hidden previously. The escape latency (the time from placing the animal in the basin until it found the platform for the first time) was measured, and the number of times a rat passed the platform was recorded.

### Statistical analysis

We performed a stepwise discriminant analysis using the SPSS v17.0 statistical package. To identify which indexes out of the nineteen considered (the body weight and sucrose preference in 6 weeks, total score of OFT, coat state score, proportion of time spent in the open arms in EPM, grooming latency of splash test, escape latency of MWM, proportion of immobility) are the most reliable as markers of the disease phenotype. In the stepwise approach adopted here, variables were entered sequentially[22].When all the variables in the model met the criterion to stay and none of the other variables met the criterion to enter, the stepwise selection process stopped [22]. The stability of the distinguishing function models was tested by cross validation.

All data in the figures or tables are presented as mean ± SD.One-way repeated measures ANOVA was performed to study the effect of body weight and sucrose preference(with the experiment of 6 week as repeated factor), with environment (control vs. CUMS) as the main factor, followed by a Fisher post-hoc analysis when required (i.e., p < 0.05). One-way ANOVA with environment (control *vs*. CUMS) as the main factor was used for other ethological data. Relationships between the every two items from the same test were evaluated by the linear Pearson correlation coefficient (r). Statistical significance was set at P < 0.05.

## Results

### Consequences of the behavioral tests

#### Body weight

**Fig 2:**
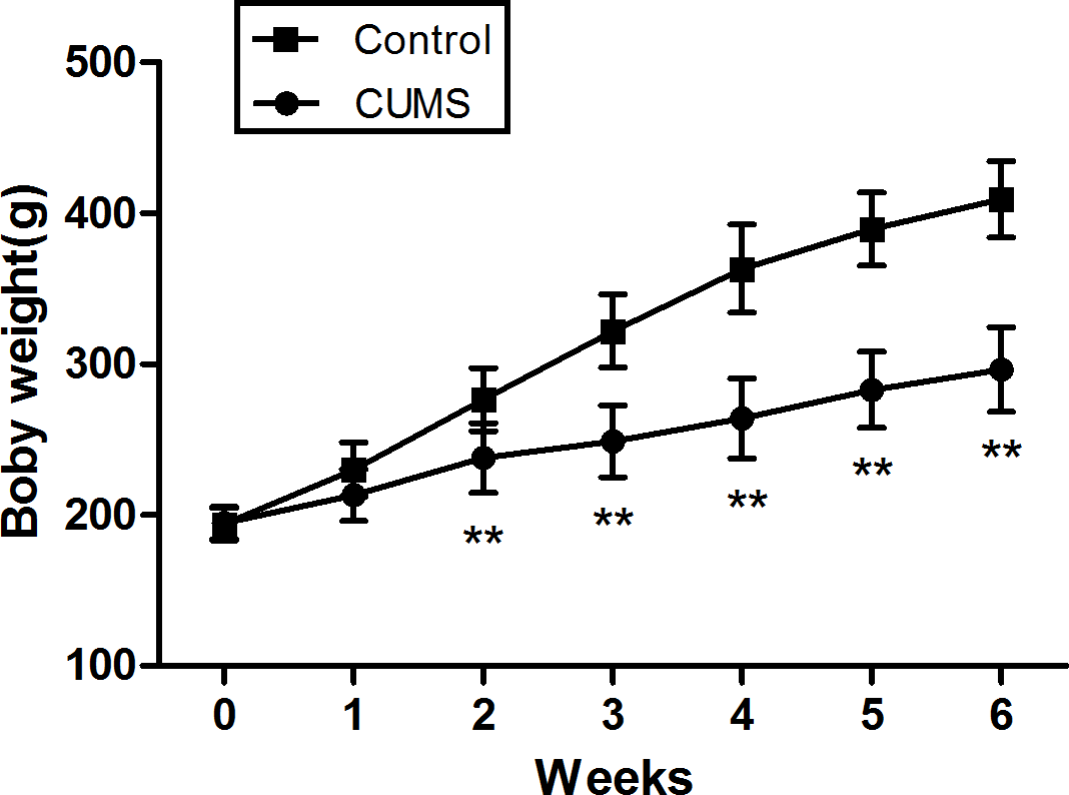
Results of the body weight of rats submitted to 6-week CUMS (mean ± SD, n = 40). **p < 0.01 vs control.

The body weight through ANOVA revealed a significant main effect on CUMS [F_(1,78)_ = 201.50, p < 0.001] and the time of per week [F_(6,468)_ = 2226.61, p < 0.001]. There is a significant interaction between the factors week and CUMS [F_(6,468)_ = 334.64, p < 0.001]. A remarkable increase in body weight with the times was observed in both groups. However, form week 2 onwards,the body weight increase significantly between CUMS-subjected and control animals. This effect was apparent after 2 week of CUMS and became highly significant from 3 week to the end.(**Fig 2**)

#### Sucrose preference test

**Fig 3:**
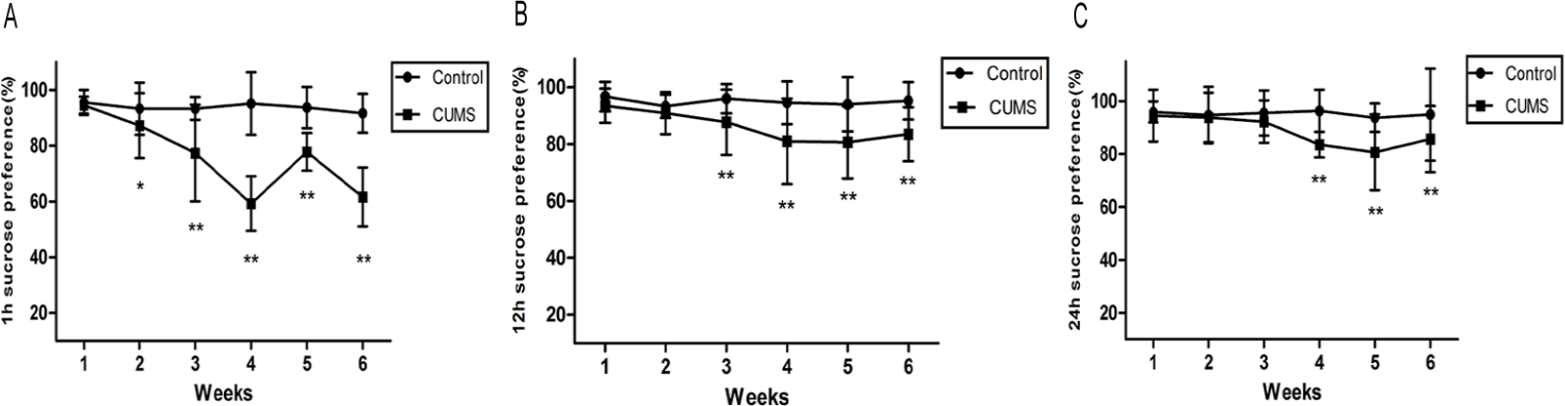
Results of sucrose preference of rats submitted to 6-week CUMS(mean ± SD, n = 40). A: 1 h sucrose preference(%); B: 12 h sucrose preference(%); C: 24 h sucrose preference(%). *p < 0.05, **p < 0.01 vs control.

In the SPT, ANOVA revealed a significant effect of CUMS (1 h: F_(1,78)_ = 414.40, p < 0.001;12 h: F_(1,78)_ = 55.12, p < 0.001; 24 h: F_(1,78)_ = 37.15, p < 0.001) and week (1 h: F_(5,390)_ = 47.70, p < 0.001; 12 h: F_(5,390)_ = 42.59, p < 0.001; 24 h: F_(5,390)_ = 17.88, p < 0.001) and a significant interaction between the factors week and CUMS(1 h: F_(5,390)_ = 41.23, p < 0.001; 12 h: F_(5,390)_ = 30.95, p < 0.001; 24 h: F_(5,390)_ = 12.60, p< 0.001). Sucrose preference was similar between the two groups at the beginning of the experiment. During the whole CUMS procedure, the sucrose preference in control rats were relatively stable even with some fluctuation, while the CUMS group appeared a marked reduction from week 2 (93.26 ± 9.39% *vs* 87.19 ± 11.60%, P < 0.05) to the end (week 6: 91.61 ± 6.98% *vs* 61.60 ± 10.53%, P < 0.01) in 1 h SPT (**Fig 3A**). Although the sucrose preference showed a decrease at 12 h and 24 h (**Fig 3B and 3C**, P < 0.01), the most significant effect was observed at 1 h.

#### Coat status test

**Fig 4:**
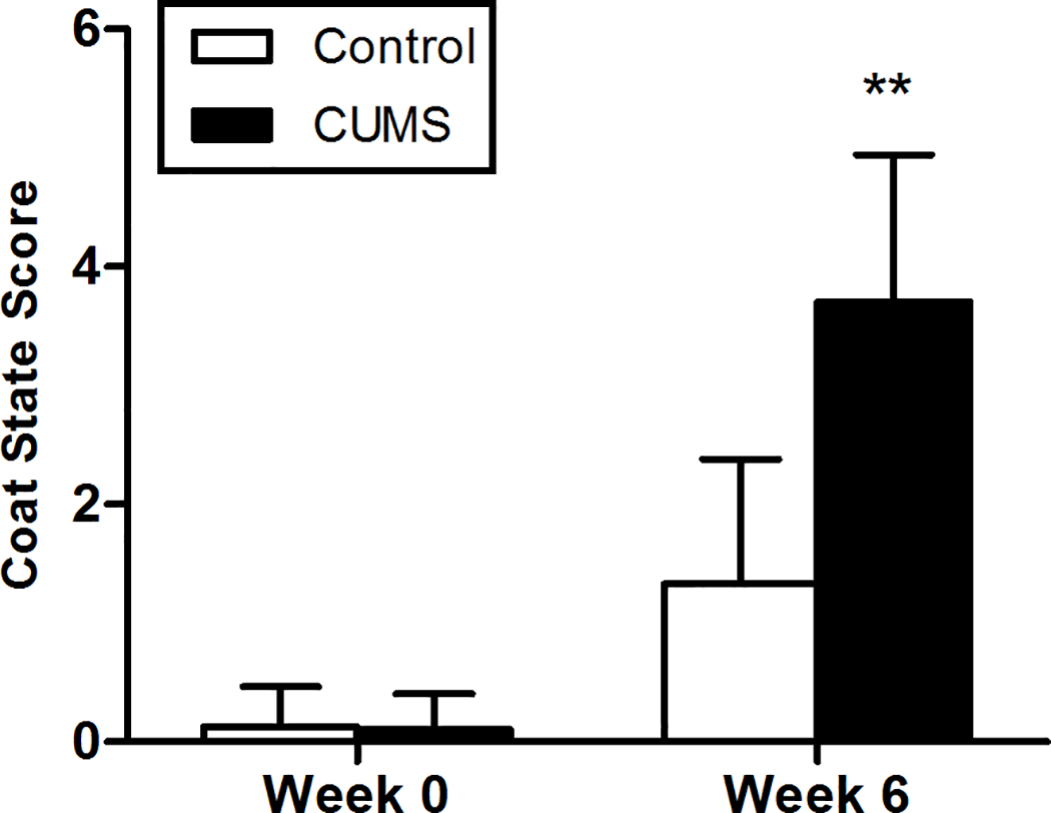
Results of the coat status test of rats submitted to 6-week CUMS (mean ± SD, n = 40). **p < 0.01 vs control.

At the end of the 6-week CUMS regimen, the results of the coat state revealed that rats subjected to the CUMS protocol exhibited a significantly decrease of self-care behavior compared to the control group. (**Fig 4**, p < 0.01)

#### Open-field test

The latency of locomotion in the CUMS group was significantly higher (p < 0.01) than that in the control group, whereas horizontal movement (p < 0.01), rearing (p < 0.01), and total scores (p < 0.01) were clearly lower. However, no significant effect was obtained for the grooming and defecating behaviors (**Table 2**).

**Table 2:** Results of horizontal movement, rearing, grooming behaviors, feces, latency (sec), total scores in the open-field test of rats submitted to 6-week CUMS (mean± SD, n = 40).

	Control	CUMS
**Latency (sec)**	1.63 ± 0.77	8.15 ± 4.30**
**Horizontal movement**	87.50 ± 10.25	20.23 ± 6.52**
**Rearing**	19.38 ± 5.87	2.80 ± 3.55**
**Grooming behaviors**	0.70 ± 0.82	0.75 ± 1.32
**Feces**	5.73 ± 5.76	6.13 ± 3.93
**Total scores**	113.30 ± 11.74	29.90 ± 11.99**

**p < 0.01 *vs* control.

#### Elevated plus-maze test

**Fig 5:**
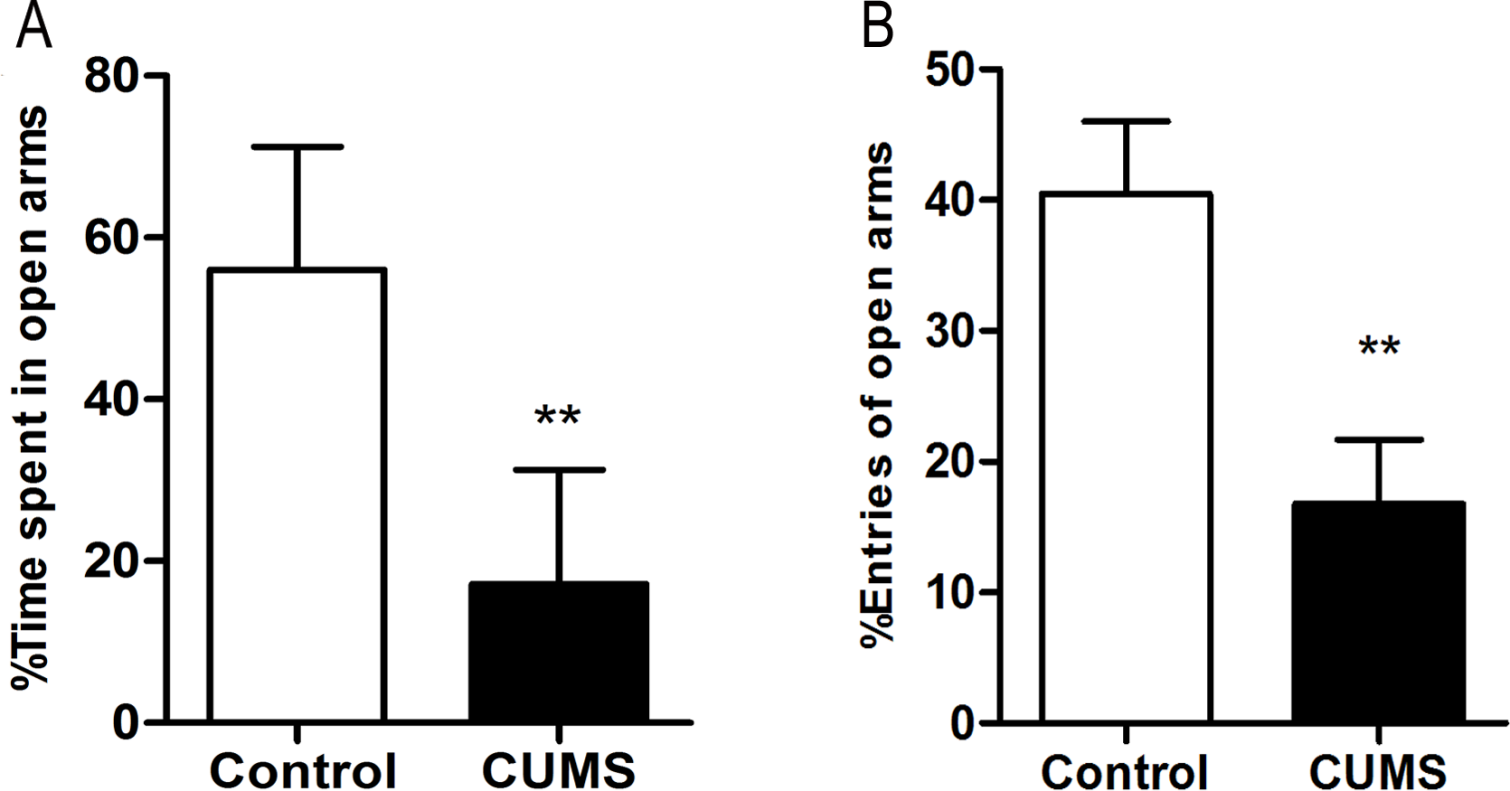
Results of the percentage of time and entries in the open arms in elevated plus-maze test of rats submitted to 6-week CUMS (mean ± SD, n = 40). A: Time spent in open arms(%); B: Entries of open arms(%).**p < 0.01 vs control.

In the EPM, CUMS group spent a significantly lower percentage of time (p < 0.01) in the open arms than control group (Fig 5A), indicative of a higher state of anxiety. The entries of the open arms (p < 0.01) was also significantly reduced after 6-week CUMS regimen in rats. (**Fig 5B**).

#### Splash test

**Fig 6:**
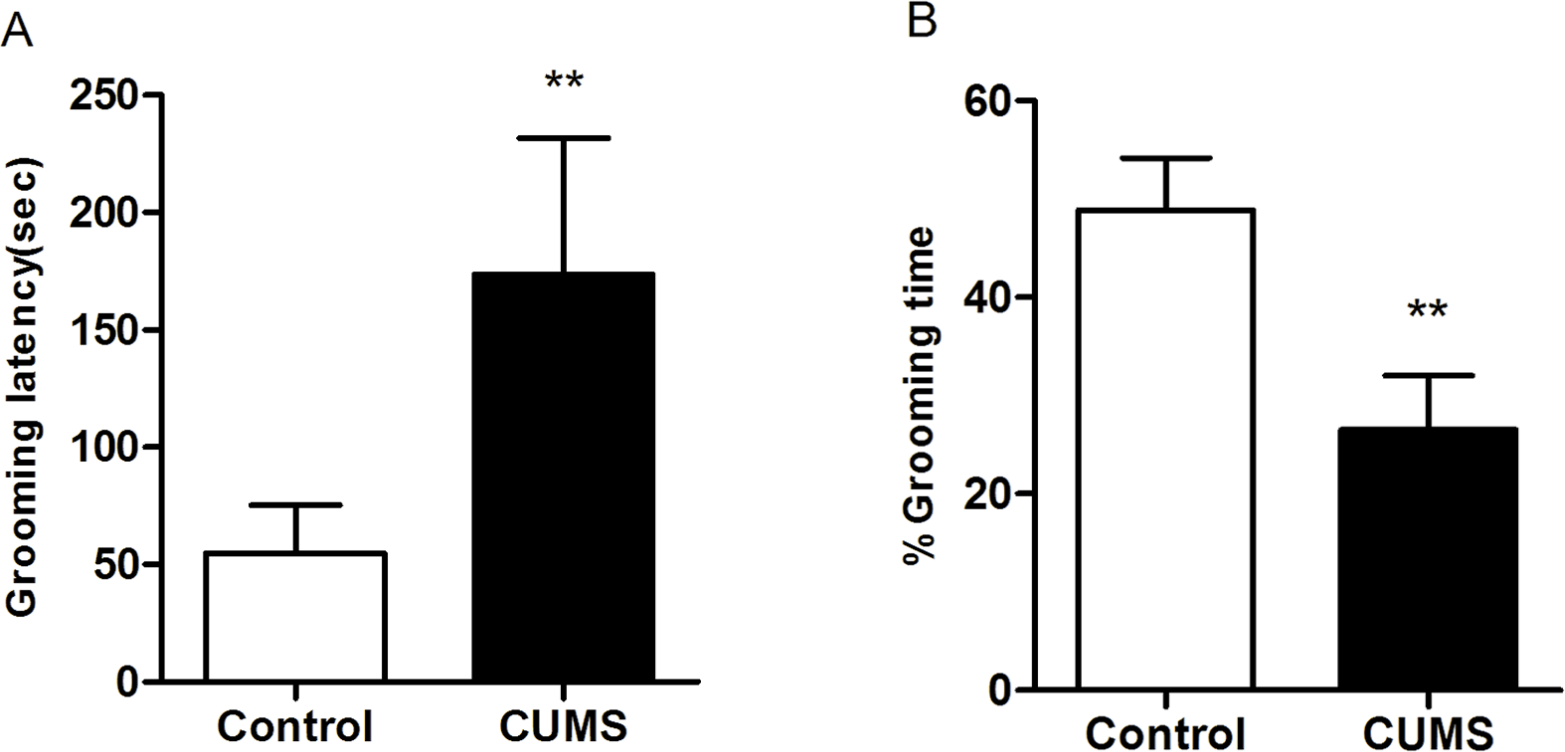
Results of the latency and time of grooming behavior following a 10% sucrose solution spray in the splash test of rats submitted to 6-week CUMS(mean ± SD, n = 40).A: Grooming latency(sec); B: Grooming time(%). **p < 0.01 vs control.

The data presented in **Fig 6** demonstrated that rats subjected to the CUMS protocol neglected coat grooming when compared to the control group. This was illustrated by increased latency (**Fig 6A**, p < 0.01) and decreased time (**Fig 6B**, p < 0.01) of grooming behavior.

#### Forced swimming test

**Fig 7:**
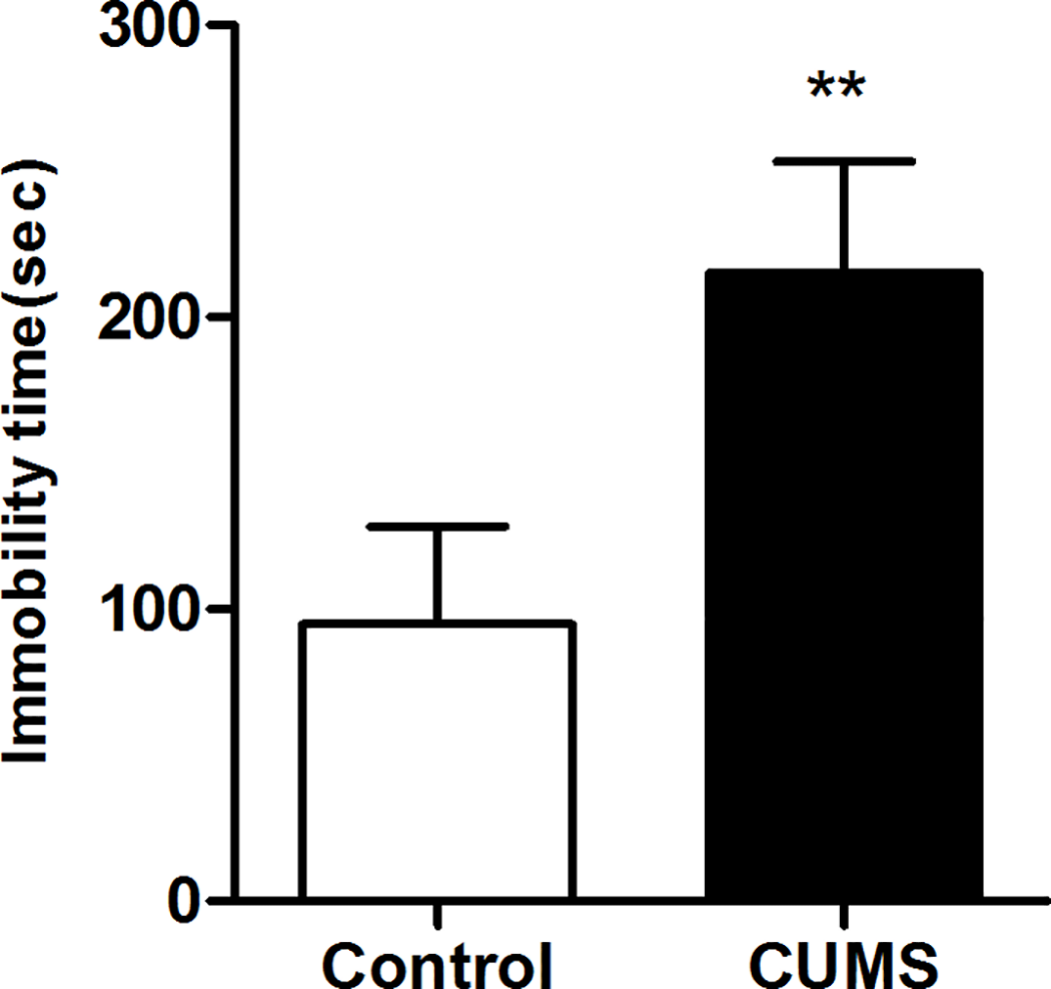
Results of forced swimming test of rats submitted to 6-week CUMS (mean ± SD, n = 40). **p < 0.01 vs control.

In the FST,there is a significant increase of the immobility time was obtained in the CUMS group(p < 0.01) that compared to the control group(**Fig 7**).

#### Morris water maze test

**Fig 8:**
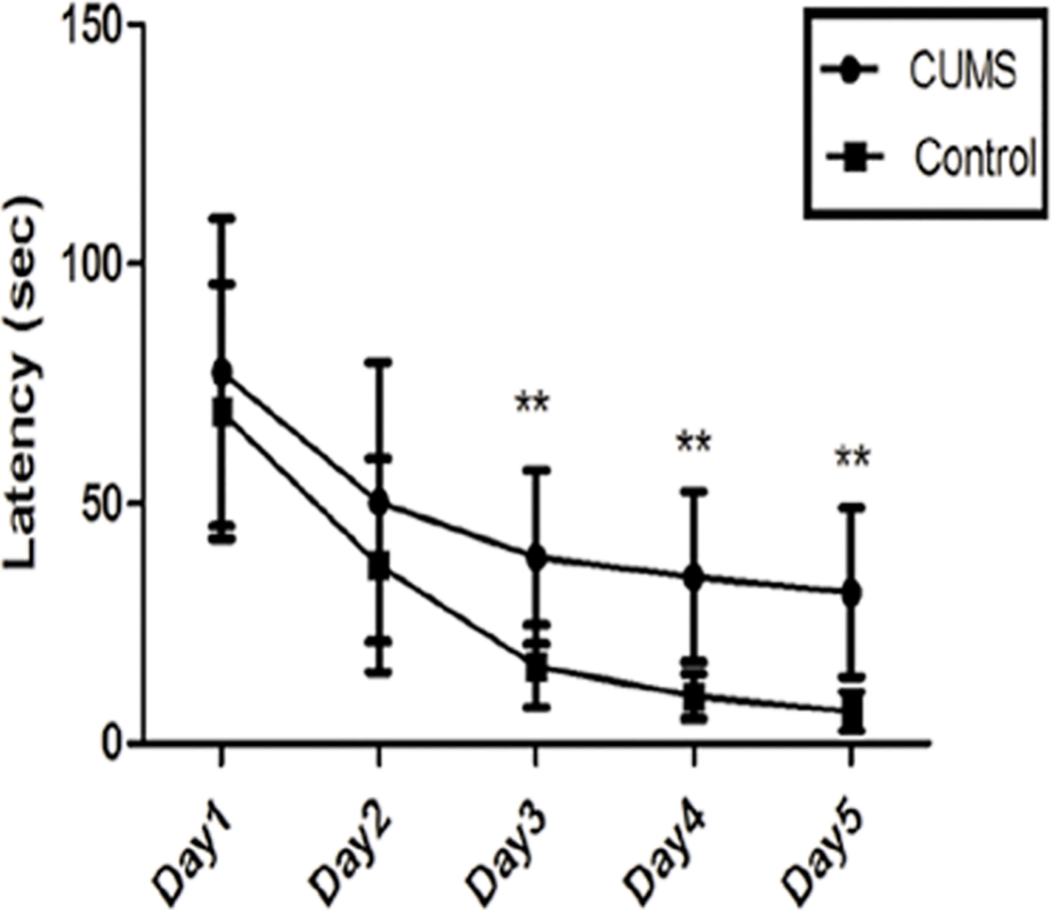
Results of latency of Morris water maze test of rats in initial spatial training(mean ± SD, n = 40). **P < 0.01 vs control.

Memory/cognitive functions were tested in the MWM. The CUMS group exhibited slow learning progress measured as latency to reach the platform on day 3,4, 5(n = 40, P < 0.01) (**Fig 8**) and decrease in the number of crosses in the MWM (n = 40,P < 0.01) compared with control group (**Table 3**).

**Table 3:** Results of the latency and the times of passing in the MWM test of rats submitted to 6-week CUMS (mean ± SD, n = 40).

	Control	CUMS
**Latency (sec)**	6.6 ± 4.08	31.48 ± 17.67**
**Times of passing**	10.50 ± 2.24	6.43 ± 1.34**

**p < 0.01 *vs* control

#### Stepwise discriminant analysis of the ethological indexes

The total sample size was 80 (CUMS group = 40, Control group = 40). The variables were separately numbered as following: N1(CUMS group = 1, Control group = 2); N2-N8(the body weight from week 0 to week 6); N9-N14(the sucrose preference from week 1 to week 6); N15(coat state score); N16(total score of OFT);N17(percentage of time spent in the open arms);N18(grooming latency of splash test);N19(percentage of immobility in FST); N20(escape latency of MWM).

In **Table 4**, eight variables out of the total 19 are reported together with their inclusion parameters, as only these eight indexes seemed to have a significant influence on identifying depression rats. Data from only the last step of the inclusion process are presented.

**Table 4:** Variables entered and inclusion parameters.

Variables entered	Code	Partial R-Square	F Value	Pr > F	Wilks' Lambda	Pr< Lambda
**Total score of OFT**	N16	0.948	38.571	< .0001	0.043	< .0001
**Body weight(week 6)**	N8	0.309	17.230	< .0001	0.035	< .0001
**Grooming latency of splash test**	N18	0.894	9.497	< .0001	0.032	< .0001
**Coat state score**	N15	0.838	13.051	< .0001	0.033	< .0001
**Proportion of immobility in FST**	N19	0.864	11.704	< .0001	0.032	< .0001
**1h sucrose preference(week 5)**	N13	0.927	6.812	< .0001	0.030	< .0001
**Body weight(week 2)**	N4	0.311	5.845	< .0001	0.030	< .0001
**1h sucrose preference(week 4)**	N12	0.956	4.106	< .0001	0.029	< .0001

In **Table 5**, we defined the CUMS group and normal group as 1 and 2 categories with 40 samples in each class. Each variable discriminatingly contributed to the cases can be reflected by the standardized discriminant function coefficient via the following two function.

From the data, the highest contribution could be ascribed to the coat state, the next were the sucrose preference and body weight.

**Table 5:** Standardized discriminant function coefficients.

		Function	
		1	2
**Body weight(week 2)**	N4	0.100	-0.167
**Body weight(week 6)**	N8	0.353	0.707
**1h sucrose preference(week 4)**	N12	0.428	0.697
**1h sucrose preference(week 5)**	N13	1.363	1.878
**Coat state score**	N15	2.288	-2.146
**Total score of OFT**	N16	0.089	0.697
**Grooming latency of splash test**	N18	0.060	-0.039
**Proportion of immobility in FST**	N19	0.183	0.050

In **Table 6**, the results of the cross validation show that the analysis correctly allocated 100% of the rats to their true group. The correct assignment rate reached 100% in the two discriminant functions, demonstrating that the two functions are stable.

**Table 6:** The assessment effect of discriminant functions.

	Group	Predicted group membership		
		Control	CUMS	Total
**Count** **%**	Control	40	1	40
	CUMS	0	40	40
	Control	100	0	100
	CUMS	0	100	100

#### Interrelationship analysis of the ethological indexes

The Pearson correlation coefficients of various ethological indexes in CUMS group are listed in **Table 7**. The variable X1 to X9 separately refer to these indexes: X1: the rearing in OFT; X2: the horizontal movement in OFT; X3: the total score of OFT; X4: the proportion of entries into the open arms; X5: the proportion of time spent in the open arms; X6: the latency in the splash test; X7: the proportion of time spent in grooming in the splash test; X8: the latency in the MWM test; X9: the times of passing in the MWM test.

**Table 7:** Results of Pearson correlation coefficients (N = 40, Prob > |r| under H0: Rho = 0).

	X1	X2	X3	X4	X5	X6	X7	X8	X9
**X1**	**1.000**	**0.643**	**0.709**	**0.332**	**0.326**	**0.036**	**0.193**	**-0.030**	**0.056**
		**<0.001**	**<0.001**	**0.036**	**0.040**	**0.826**	**0.233**	**0.853**	**0.731**
**X2**		**1.000**	**0.972**	**0.347**	**0.418**	**0.235**	**-0.148**	**-0.025**	**-0.020**
			**<0.001**	**0.028**	**0.007**	**0.144**	**0.362**	**0.877**	**0.902**
**X3**			**1.000**	**0.373**	**0.411**	**0.181**	**-0.129**	**-0.034**	**0.035**
				**0.018**	**0.008**	**0.263**	**0.426**	**0.834**	**0.832**
**X4**				**1.000**	**0.782**	**0.316**	**0.039**	**-0.042**	**-0.006**
					**<0.001**	**0.047**	**0.813**	**0.797**	**0.971**
**X5**					**1.000**	**0.336**	**0.165**	**-0.191**	**0.065**
						**0.034**	**0.310**	**0.238**	**0.691**
**X6**						**1.000**	**-0.003**	**0.140**	**-0.142**
							**<0.984**	**0.387**	**0.382**
**X7**							**1.000**	**-0.041**	**0.169**
								**0.802**	**0.297**
**X8**								**1.000**	**-0.331**
									**<0.037**
**X9**									**1.000**

It is obviously from **Table 7** that in the OFT, the rearing (X1) and horizontal movement (X2) had a medium interrelationship (X1, X2: r = 0.643, p < 0.001). Between the total score (X3)and rearing (X1) or horizontal movement (X2),there was a strong interrelationship(X1, X3: r = 0.709, p < 0.001; X2, X3: = 0.972, p < 0.001).

On the other hand, the number of entries into the open arms (X4*)* and the time spent in the open arms (X5) in EPM had a strong interrelationship (r = 0.782, p < 0.001).

As for other data, the Pearson correlations coefficients were not significant.

## Discussion

In the present study, we aimed to establish the CUMS rat model and find the interrelationships among the behavioral tests. The CUMS procedure was developed as an animal model of depression that mimicked the time course and behavioral changes required for investigating the effects of chronic drug treatments [3,23]. In addition to the major characteristics [3] such as decrease in body weight [24,25], this paradigm replicates many other behavioral disturbances seen in depression. While certain studies [9,26,27] showed CUMS-induced decrease in self-care and grooming, which could be interpreted as a loss of motivational behavior, considered to parallel another important symptom of depression [6], other studies reported that CUMS was able to dramatically increase the immobility time in FST [11,14,28], which was later confirmed as “behavioral despair” [29]. The OFT was designed to assess the locomotor activity of rodents, which was used as a behavioral endpoint by Katz and colleagues [30]. Animals displayed decreased locomotor activity in OFT after CUMS [18,31], which indicates the loss of exploration and interest, two instinctive activities of normal animals in a novel environment [32]. Meanwhile, other researchers observed that anxiety [12,33] and memory deficits [14,34] were induced by CUMS, showing the conflicting emotions between fear induced by novelty and exploratory behavior [35] and impairments in cognitive function, respectively.

In the present study, we first tried to establish a CUMS-induced rat model with depression-like behavioral changes by performing eight most frequently used behavioral tests. In our study, we observed a significant decrease in body weight, a remarkable reduction in 1 h sucrose preference, a deterioration of coat state, and a notable decrease in grooming behaviors in the splash test. Moreover, CUMS rats also had a protracted latency of locomotion and a significant decrease in horizontal and vertical movement in OFT, a prominent reduction of entries and time in the open arms in EPM, an arresting increase in the duration of immobility in FST, and cognitive dysfunction in MWM. It is worth mentioning this in view of the current reports related to SPT [3,11,18,31]. As there are not any unified standards of sucrose solution concentration and time points for testing liquid consumption, we fixed the concentration at 1% (suggested by Paul Willner) and set up 3 time points to measure the consumption in our study, which were at 1 h, 12 h, and 24 h after providing the liquid. Our results showed that at the 1-h interval, the sucrose preference of CUMS rats was significantly different from that of the control rats. At the intervals of 12 h and 24 h, the rats subjected to CUMS took significantly more time through SPT. These data suggest that measuring sucrose consumption at 1 h is most effective in SPT. In our opinion, such a unique conclusion could be attributed to several parameters of the housing conditions, test timing, and protocol, as well as the strain of rat chosen. All the above results strongly suggested that our current protocol of a 6-week-long CUMS successfully replicated the behavioral changes paralleling the clinical symptoms and accompanying symptoms of major depression.

It is clear that multiple factors influence the stress response. Generally, researchers use several behavioral endpoints when assessing depression-like phenotypes for garnering a depressive animal model with validity. As the purpose of our study was to determine certain criteria for combining these behavioral endpoints, we analyzed the interrelationships among behavioral indexes through stepwise discriminant analysis. Discriminant analysis is a multivariate dimension-reduction technique, whose main objective is to draw out a set of linear combinations of the quantitative variables (discriminant functions) that best account for the differences among the treatment groups [36]. Following the stepwise discriminant analysis, we observed that the body weight change, percentage of sucrose preference, coat stat score, open-field score, grooming latency of splash test, and immobility time in FST were the most relevant tests for the rat model of CUMS (in descending order of the contributions to the discriminant function equations). The other tests showed a poor relevance to the CUMS rats, which can be a reference for future depression research. The analysis allows us to evaluate the contribution of each variable in discriminating among groups, thus, representing an optimal selection tool for the most informative prognosticators in animal depression model. In our study, the following points are worth mentioning. (1) Incorporating all the 6-week data of body weight and sucrose preference leads to complicacy in the discriminant models. However, the body weight of weeks 2 and 6, together with the sucrose preference in weeks 4 and 5, demonstrated the development process of depression have the characteristic of periodic change. (2) Most current studies [11,18,28,31] shows that investigators prefer to take body weight, SPT, OFT, and FST to measure behavioral endpoints in model animals, which is consistent with our discriminant analysis result. Anhedonia, the major endpoint of CUMS model [3], is associated with other analogues of depressive symptoms, such as increased floating duration in FST and decreased exploration of novelty in OFT. The comprehensiveness and relevance of these indexes greatly improve the superiority of methods. (3) Coat state and splash test both connect with self-care behavior and they are all incorporated into the discriminant models, which reveal that apathy is a crucial characteristic of depression [6]. It might be possible to establish coat state or splash test as estimates of self-care behavior in the future, which requires further proof.

The Pearson correlation coefficients revealed that the total score was related to horizontal movement and rearing. Moreover, the relationship between total score and horizontal crossing is stronger than that between total score and rearing. Also considering the fact that no significant effect was obtained for grooming and defecating behaviors in OFT (Table 2), it is suggested that the CUMS procedure is more likely to weaken the locomotion activity than the automatic nervous system function of rats, and horizontal movement might represent the change in locomotor activity to some extent. On the other hand, the time spent in the open arms and the number of entries into the open arms in EPM had a strong correlation, which suggests that we could measure only one ethological index (either the time spent in the open arms or the number of entries into the open arms) instead of both in EPM. As for our data, there was only a slight or no correlation between them, and this result might help in the research on depression models in future experiments. Analysis by stepwise discriminant and Pearson correlation coefficient might contribute to the study of depressive models in future experiments. This can achieve two things at once, building a reliable CUMS model and improving the work efficiency.

In conclusion, our current protocol of a 6-week-long CUMS is able to induce depression-like behavioral changes characterized by anhedonia, lost weight, apathy, behavioral despair, psychomotor retardation, anxiety, and cognitive dysfunction. The comparatively effective endpoints, i.e., body weight change, percentage of sucrose preference, coat stat score, open-field score, grooming latency of splash test, and immobility time in FST, are screened because of their close association, and their mutual interrelationships and reciprocal complementation might be advantageous to assess the CUMS-induced depression model. The strong interrelationships among indexes in OFT and EPM helped discard some unnecessary indexes, and it might avoid wastage of expense and labor. However, the result of discriminant analysis might exhibit deviation due to the limited number of animal samples. Accordingly, further studies are needed with a greater number of samples and newer analysis methods to confirm our discovery.

## Supporting information

S1 Appendix The statistical analysis of relevant data. (XLSX)

## References

[1] Kessler RC, Birnbaum H, Bromet E, Hwang I, Sampson N, Shahly V. Age differences in major depression: results from the National Comorbidity Survey Replication (NCS-R). Psychol Med. 2010; 40(2): 225–37. doi: 10.1017/S0033291709990213 19531277 10.1017/S0033291709990213 PMC2813515

[2] Yiend J, Paykel E, Merritt R, Lester K, Doll H, Burns T. Long term outcome of primary care depression. J Affect Disord. 2009; 118(1–3): 79–86. doi: 10.1016/j.jad.2009.01.026 19246103 10.1016/j.jad.2009.01.026

[3] Willner P, Towell A, Sampson D, Sophokleous S, Muscat R. Reduction of sucrose preference by chronic unpredictable mild stress, and its restoration by a tricyclic antidepressant. Psychopharmacology (Berl). 1987; 93(3): 358–64. 3124165 10.1007/BF00187257

[4] American Psychiatric Association, Task Force on DSM-IV, 2000. Diagnostic and Statistical Manual of Mental Disorders: DSM-IV-TR, 4th, text revision ed. American Psychiatric Association, Washington, DC, 2000.

[5] Willner P. Animal models of depression: validity and applications. Adv Biochem Psychopharmacol. 1995; 49: 19–41. 7653333

[6] Willner P. Chronic mild stress (CMS) revisited: consistency and behavioural-neurobiological concordance in the effects of CMS.Neuropsychobiology. 2005; 52(2): 90–110. doi: 10.1159/000087097 16037678 10.1159/000087097

[7] Gonda X, Pompili M, Serafini G, Carvalho AF, Rihmer Z, Dome P. The role of cognitive dysfunction in the symptoms and remission from depression, Ann. General Psychiatry. 2015; 14:27. 10.1186/s12991-015-0068-9 PMC4578787 26396586

[8] Tondo L, Vázquez GH, Baldessarini RJ. Options for pharmacological treatment of refractory bipolar depression, Curr. Psychiatry Rep. 2014; 16(2): 431 doi: 10.1007/s11920-013-0431-y 24425269 10.1007/s11920-013-0431-y

[9] Luo Y, Kuang S, Li H, Ran D, Yang J. cAMP/PKA-CREB-BDNF signaling pathway in hippocampus mediates cyclooxygenase 2-induced learning/memory deficits of rats subjected to chronic unpredictable mild stress. Oncotarget. 2017; 8(22): 35558–35572. doi: 10.18632/oncotarget.16009 28415673 10.18632/oncotarget.16009 PMC5482598

[10] Luo Y, Kuang S, Xue L, Yang J. The mechanism of 5-lipoxygenase in the impairment of learning and memory in rats subjected to chronic unpredictable mild stress. Physiol Behav. 2016; 167: 145–153. doi: 10.1016/j.physbeh.2016.09.010 27640130 10.1016/j.physbeh.2016.09.010

[11] Luo DD, An SC, Zhang X. Involvement of hippocampal serotonin and neuropeptide Y in depression induced by chronic unpredicted mild stress. Brain Res Bull. 2008; 77(1): 8–12. doi: 10.1016/j.brainresbull.2008.05.010 18579108 10.1016/j.brainresbull.2008.05.010

[12] Qi X, Lin W, Li J, Pan Y, Wang W. The depressive-like behaviors are correlated with decreased phosphorylation of mitogen-activated protein kinases in rat brain following chronic forced swim stress. Behav Brain Res. 2006; 175(2): 233–40. doi: 10.1016/j.bbr.2006.08.035 17050000 10.1016/j.bbr.2006.08.035

[13] Yalcin I, Aksu F, Belzung C. Effects of desipramine and tramadol in a chronic mild stress model in mice are altered by yohimbine but not by pindolol. Eur J Pharmacol. 2005; 514(2–3): 165–74. doi: 10.1016/j.ejphar.2005.03.029 15910803 10.1016/j.ejphar.2005.03.029

[14] Burda K, Czubak A, Kus K, Nowakowska E, Ratajczak P, Zin J. Influence of aripiprazole on the antidepressant, anxiolytic and cognitive functions of rats.Pharmacol Rep. 2011; 63(4): 898–907. 22001977 10.1016/s1734-1140(11)70605-3

[15] Ortmann CF, Réus GZ, Ignácio ZM. Enriched Flavonoid Fraction from Cecropia pachystachya Trécul Leaves Exerts Antidepressant-like Behavior and Protects Brain Against Oxidative Stress in Rats Subjected to Chronic Mild Stress. Neurotox Res.2016; 29(4): 469–83 doi: 10.1007/s12640-016-9596-6 26762362 10.1007/s12640-016-9596-6

[16] Mutlu O, Gumuslu E, Ulak G, Celikyurt IK, Kokturk S, Kır HM, et al Effects of fluoxetine, tianeptine and olanzapine on unpredictable chronic mild stress-induced depression-like behaviorin mice. Life Sci 2012; 91: 1252–1262 doi: 10.1016/j.lfs.2012.09.023 23069580 10.1016/j.lfs.2012.09.023

[17] Isingrini E, Camus V, Le Guisquet AM, Pingaud M, Devers S, Belzung C. Association between repeated unpredictable chronic mild stress (UCMS) procedures with a high fat diet: a model of fluoxetine resistance in mice. PLoS ONE 2010; 5(4): e10404 doi: 10.1371/journal.pone.0010404 20436931 10.1371/journal.pone.0010404 PMC2861008

[18] Lin YH, Liu AH, Xu Y, Tie L, Yu HM, Li XJ. Effect of chronic unpredictable mild stress on brain-pancreas relative protein in rat brain and pancreas. Behav Brain Res. 2005; 165(1): 63–71. doi: 10.1016/j.bbr.2005.06.034 16154211 10.1016/j.bbr.2005.06.034

[19] Mehta V1, Parashar A2, Udayabanu M3. Quercetin prevents chronic unpredictable stress induced behavioral dysfunction in mice by alleviating hippocampal oxidative and inflammatory stress. Physiol Behav. 2017; 171: 69–78. doi: 10.1016/j.physbeh.2017.01.006 28069457 10.1016/j.physbeh.2017.01.006

[20] Słupski W, Trocha M, Rutkowska M. Pharmacodynamic and pharmacokinetic interactions between simvastatin and diazepam in rats. Pharmacol Rep. 2017; 69(5): 943–952 doi: 10.1016/j.pharep.2017.03.012 28666152 10.1016/j.pharep.2017.03.012

[21] Lino-de-Oliveira C, De Lima TC, de Pádua Carobrez A. Structure of the rat behaviour in the forced swimming test. Behav Brain Res. 2005; 158(2): 243–50. doi: 10.1016/j.bbr.2004.09.004 15698890 10.1016/j.bbr.2004.09.004

[22] Ciani E, Alloggio I, Petazzi F, Pieragostini E. Looking for prognosticators in ovine anaplasmosis: discriminant analysis of clinical and haematological parameters in lambs belonging to differently susceptible breeds experimentally infected with Anaplasma ovis. Acta Vet Scand. 2013; 55:71 doi: 10.1186/1751-0147-55-71 24053615 10.1186/1751-0147-55-71 PMC3850957

[23] Willner P, Muscat R, Papp M. Chronic mild stress-induced anhedonia: a realistic animal model of depression. Neurosci Biobehav Rev. 1992; 16(4): 525–34. 1480349 10.1016/s0149-7634(05)80194-0

[24] Matthews K, Forbes N, Reid IC. Sucrose consumption as an hedonic measure following chronic unpredictable mild stress. Physiol Behav. 1995; 57(2): 241–8. 7716198 10.1016/0031-9384(94)00286-e

[25] Willner P, Moreau JL, Nielsen CK, Papp M, Sluzewska A. Decreased hedonic responsiveness following chronic mild stress is not secondary to loss of body weight. Physiol Behav. 1996; 60(1): 129–34. 8804652 10.1016/0031-9384(95)02256-2

[26] Surget A and Belzung C. Unpredictable chronic mild stress in mice In: Kalueff A.V. and LaPorte J.L., editors. Experimental Animal Models in Neurobehavioral Research, New York: Nova Science, pp. 2009; 79–112.

[27] Surget A, Saxe M, Leman S, Ibarguen-Vargas Y, Chalon S, Griebel G, et al Drug-dependent requirement of hippocampal neurogenesis in a model of depression and of antidepressant reversal. Biol Psychiatry. 2008; 64(4): 293–301. doi: 10.1016/j.biopsych.2008.02.022 18406399 10.1016/j.biopsych.2008.02.022

[28] Vitale G, Ruggieri V, Filaferro M, Frigeri C, Alboni S, Tascedda F, et al Chronic treatment with the selective NOP receptor antagonist [Nphe 1, Arg 14, Lys 15]N/OFQ-NH 2 (UFP-101) reverses the behavioural and biochemical effects of unpredictable chronic mild stress in rats.Psychopharmacology (Berl). 2009; 207(2): 173–89. 19711054 10.1007/s00213-009-1646-9

[29] Nishimura H, Tsuda A, Oguchi M, Ida Y, Tanaka M. Is immobility of rats in the forced swim test "behavioral despair"? Physiol Behav. 1988; 42(1): 93–5. 3387484 10.1016/0031-9384(88)90266-1

[30] Katz RJ. Animal model of depression: pharmacological sensitivity of a hedonic deficit. Pharmacol Biochem Behav. 1982; 16(6): 965–8. 7202217 10.1016/0091-3057(82)90053-3

[31] Jiang P, Zhang WY, Li HD, Cai HL, Liu YP, Chen LY. Stress and vitamin D: altered vitamin D metabolism in both the hippocampus and myocardium of chronic unpredictable mild stress exposed rats. Psychoneuroendocrinology. 2013; 38(10): 2091–8. doi: 10.1016/j.psyneuen.2013.03.017 23608137 10.1016/j.psyneuen.2013.03.017

[32] Willner P. Validity, reliability and utility of the chronic mild stress model of depression: a 10-year review and evaluation. Psychopharmacology (Berl). 1997; 134(4): 319–29. 10.1007/s002130050456 9452163

[33] Guo JY, Bian H, Yao Y. Chronic unpredictable mild stress induces parallel reductions of 15-PGDH in the hypothalamus and lungs in rats. Behav Brain Res. 2015; 286: 278–84. doi: 10.1016/j.bbr.2015.03.013 25779923 10.1016/j.bbr.2015.03.013

[34] Liu D, Zhang Q, Gu J, Wang X, Xie K, Xian X. Resveratrol prevents impaired cognition induced by chronic unpredictable mild stress in rats. Prog Neuropsychopharmacol Biol Psychiatry. 2014; 49: 21–9. doi: 10.1016/j.pnpbp.2013.10.017 24184538 10.1016/j.pnpbp.2013.10.017

[35] Montgomery KC. The relation between fear induced by novel stimulation and exploratory behavior. J Comp Physiol Psychol. 1955; 48(4): 254–60. 13252152 10.1037/h0043788

[36] Carey G,Multivariate analysis of variance (MANOVA) II: practical guide to ANOVA and MANOVA for SAS.Retrieved from http://ibgwww.colorado.edu/~carey/p7291dir/handouts/manova2.pdf

